# Alteration of Gut Microbiome and Correlated Amino Acid Metabolism Contribute to Hyperuricemia and Th17-Driven Inflammation in *Uox*-KO Mice

**DOI:** 10.3389/fimmu.2022.804306

**Published:** 2022-02-07

**Authors:** Siyue Song, Yu Lou, Yingying Mao, Xianghui Wen, Moqi Fan, Zhixing He, Yang Shen, Chengping Wen, Tiejuan Shao

**Affiliations:** ^1^ College of Basic Medical Sciences, Zhejiang Chinese Medical University, Hangzhou, China; ^2^ School of Public Health, Zhejiang Chinese Medical University, Hangzhou, China; ^3^ The Second Clinical Medical College, Zhejiang Chinese Medical University, Hangzhou, China

**Keywords:** gut microbiota, hyperuricemia (HUA), Gout, amino acid metabolism, T cell immunity

## Abstract

Although gut dysbiosis had been demonstrated to be an important factor affecting hyperuricemia (HUA) and gout, little is known for its potential mechanistic connections. In this study, *Uox*-KO mice model that with spontaneously developed pronounced HUA and urate nephropathy was used to explore the pathophysiologic mechanism of microbiota alterations in HUA and gout with integrated multi-omics analysis. 16S rRNA gene sequencing was performed to characterize the characteristic bacteria, and untargeted LC/MS analysis was applied to reveal the featured metabolites. Our results showed there was a significant shift in gut microbiota composition and function in *Uox*-KO mice compared to WT mice and apparent metabolomics differences between the two groups. Among them, amino acids metabolism appears to play a critical role. Correlation analysis further revealed that the characteristic metabolites were strongly influenced by the discrepant bacterial genera. Furthermore, impairment of intestinal integrity and profound alterations in the profile of solute carrier family resulted in dysregulation of amino acids transportation, which subsequently impacted serum uric acid level and CD4^+^ Th17 driven inflammation. Together, these data indicate that gut dysbiosis promotes purine metabolism disorder and inflammation in *Uox*-KO mice. Remodeling the gut microbiota is a promising strategy to combat HUA and gout.

## Introduction

Hyperuricemia (HUA) is a metabolic disorder caused by abnormal uric acid (UA) metabolism, and it has become the second most common metabolic disease with changes in lifestyle and dietary patterns (1). Gout is the most common complaint associated with HUA (2). Uncontrolled HUA and gout can cause significant joint and organ damage (3). It also promotes diabetes, metabolic syndrome, atherosclerosis, chronic kidney disease, and cardiovascular disease, increasing the public health burden and can be life-threatening in severe cases (4).

Although HUA and associated gout have been mainly defined as the result of insufficient kidney UA excretion, emerging evidence highlights the importance of the intestinal tract and colonized gut microbiota in the development of HUA and gout (5). Several transporters promoting UA secretion have been identified in the intestine (6). Gut microbiota and its metabolites have been proven to directly or indirectly participate in the metabolism of purine and UA (7–9). The complex interactions between intestinal microbiota and the host provide crucial insight into the pathogenesis of many inflammatory diseases including gout (5, 10). The intestine and gut microbiota have become a new target for the prevention and treatment of HUA and gout (11, 12).

On the other side, even though macrophages and neutrophils were considered to be the principal immune cells and the NLRP3 inflammasome was the major pathway involved in gout inflammation, recent studies emphasized an emerging role of T cell subsets in the pathogenesis of gout (13). It was reported that abnormal functions of several T cell subsets like Th17 and aberrant expressions of their signature cytokines existed in gout (14). Targeting pro-inflammatory T cells and corresponding cytokines is a promising preventive and therapeutic strategy for gout. However, the molecular and cellular mechanisms underlying the role of those T cell subsets in gout are largely elusive.

This study aims to investigate the mechanistic linkage between gut microbiota and HUA and gout. To this end, we developed a urate oxidase (*Uox*)-knockout (*Uox^–/–^*) mouse model which has been reported as a suitable model of HUA and more closely mimic purine metabolism in humans (2). We investigated the association between alteration of gut microbiota and gout, and probed the possible mechanistic links between intestinal microbiota and HUA and joint inflammation in the aspects of metabolites. We revealed that gut microbiota composition and amino acid (AA) metabolism are critical important for the catabolism of purine nucleotides and infiltration of CD4^+^ Th17 cells, which plays important roles in HUA and gouty inflammation. The elucidation of the mechanisms underlying the immune regulation by microbiota may hold promise for developing potential intervention strategies for HUA and gout patients.

## Materials And Methods

### Animals and Disease Assessment

Male mice aged 6 to 8 weeks were used. *Uox* gene-deficient (*Uox* ^-/-^, *Uox*-KO) mice on the background of C57BL/6J were obtained from Shanghai Southern Model Biotechnology Co., Ltd. Homozygous *Uox* ^-/-^ and *Uox* ^+/+^ (Wild Type, WT) mice were generated by intercrossing heterozygous mice and genotyped using a PCR-based method (**Table S1**). Under standard environmental conditions, the mice were housed in the Laboratory Animal Center of Zhejiang Chinese Medical Animal Care (AAALAC). Experimental procedures were approved by the Laboratory Animal Management and Welfare Ethical Review Committee of Zhejiang Chinese Medical University (Permission number: 20190400105).

The concentration of serum uric acid (SUA), urinary uric acid (UUA), serum creatinine (SCr), urinary creatinine (UCr), urinary total protein (UTP) and urinary albumin (U-Alb) were detected by an automatic biochemical analyzer (Hitachi #3100, Tokyo, Japan). Footpad swelling was measured, and the mechanical pain threshold was quantified as reported previously (15). The claw region of mice was analyzed with HE staining for conventional morphological evaluation and with immunohistochemistry (IHC) assay for colon tight-junction protein expression (**Table S2**). Transmission electron microscopy was used for the investigation of the functional morphology of intestinal villi.

### Co-Housing Experiment and Fecal Microbiota Transplantation

For co-housing experiment, the male offspring were separated once the gene type of the mice were determined. Separated WT and KO mice were fed adaptively for about 3 weeks until the gut bacteria of each group were settled down. The co-housed WT and KO mice were then bred in the same cage for 8 weeks, while the regular WT and *Uox*-KO mice were bred in separate cages to avoid microbiota cross transfer. For fecal microbiota transplantation (FMT), fresh feces were collected from *Uox-*KO mice, immediately placed into PBS (1×) and steeped for 1 min. Then, the dissolved feces were centrifuged at 1000 g (4°C) for 3 min. The suspension was collected and 100 μL of bacterial suspension was then delivered to each recipient mouse *via* oral gavage within 10 min for 4 weeks.

### DNA Extraction, 16S rRNA Gene Sequencing and Data Analysis

The middle colonic feces were collected, and total DNA was extracted using the QIAmp DNA microbiome kit (Qiagen). The 16S rRNA gene (V3-V4 region) was sequenced using the Illumina novaseq platform. The clean sequences were clustered into operational taxonomic units (OTUs) at 97% similarity. Raw sequences were deposited in the Sequence Read Archive database (http://www.ncbi.nlm.nih.gov/sra), with the accession numbers ranging from SAMN21847327 to SAMN21847340 and SAMN21848825 to SAMN21848852. Alpha and beta diversity were analyzed by R software (4.0.5, vegan package). The linear discriminant analysis (LDA) effect size (LEfSe) method was performed to discover taxa differentially abundant between different groups. Spearman’s correlation was used to determine the correlation between the correlation of microflora with clinical indicators in R software (4.0.5, psych package). The co-occurrence network was visualized by Cytoscape software (3.7.0). PICRUSt2 was utilized to predict functional profiles basing on Kyoto Encyclopedia of Genes and Genomes (KEGG). Pathway and network enrichment analyses of the differentially expressed SLCs were performed on Metascape (http://metascape.org) and visualized by Cytoscape software. Mental’s r was adopted to compute the relationships between the microflora, gout symptoms, CD4^+^ T and metabolite functional composition.

### Untargeted Fecal, Serum, and Kidney Metabolomics Analysis (LC-MS)

Fecal, renal, and serum metabolites were extracted with 20% methanol solution with vortex and sonication (16). All extracted metabolites were analyzed using an ultra-high pressure liquid chromatography (UPLC) coupled with a triple quadrupole time-of-flight (TOF) system (ABSCIEX-Triple TOF 5600; AB SCIEX, Framingham, MA, USA). The LC-MS analysis procedure was described in the supplementary material. MS raw data files were converted into the mzML format using ProteoWizard and then processed using R package XCMS. Differential metabolites among the two groups were summarized and mapped into their biochemical pathways through metabolic enrichment and pathway analysis based on database search (KEGG).

### Transcriptome Analysis of SLC Transporters

High-quality colonic RNA was extracted and used to construct the sequencing library. The RNA-seq library was constructed and subsequent transcriptome sequencing was performed by MajorBio Biotech Co., Ltd. (Shanghai, China) on an Illumina Hiseq X-ten sequencer. RNA-Seq data was read as described in the supplementary material (17). The differential expression was visualized by heat map using R software.

### Analysis of mRNA and Protein Expression

Total RNA was extracted from the colon with Trizol reagent (Invitrogen) and reversed transcribed into complementary DNA (cDNA) using the random hexamers primers with HiFiScript cDNA Synthesis Kit. Real-time quantitative PCR (*q*PCR) was performed using SYBR Green Premix Pro Taq HS *q*PCR Kit with Roche LightCycler**^®^** 96 SW1.1 instrument. The 2^−ΔΔCt^ method was used to calculate the relative expression level of each sample referring to internal β‐actin expression. The sequence of primers is shown in **Table S3**.

Total protein was extracted from the colon with cold RIPA lysis buffer with protease and phosphatase inhibitors. Whole protein concentrations were detected by BCA protein assay. The detailed western blotting procedure was described in the supplementary material, and the primary antibodies used were listed in **Table S4**. The protein bands were visualized using an Odyssey Infrared Imaging System (LI-COR Biosciences). Image J was used to quantify protein grey value as a ratio to β-actin.

### Measurement of Cytokines

The colon tissues were homogenized in cold PBS, and the supernatant was collected to determine the concentration of IFN-γ, TNF-α, IL-1β, IL-6, and IL-17 by ELISA kits following the manufacturer’s instructions. Serum levels of cytokines were measured as well.

### Isolation of CD4^+^ T-Cells From the Gut

The lumenal content was removed, and the sectioned intestine was washed with ice-cold PBS. The tissue was then cutted into 1-2 mm pieces and digested with 2 mg/ml Collagenase D (Cat#: 11088858001, Roche) and 2 mg/ml DNase I (Cat#: 10104159001, Sigma-Aldrich) in RPMI-1640 medium (Gibco) at 37°C for 40 min under slow rotation. The supernatants were filtered by 70 µm mesh. The collected cells were resuspended in 30% Percoll (Sigma) and carefully overlaid onto 70% Percoll. After centrifugation, the cell layers located between the two Percoll layers were collected. CD4^+^ T-cells were isolated using EasySep™ Mouse CD4 Positive Selection Kit II (Cat#: 18952, STEMCELL Technologies, Canada).

### Flow Cytometry Analysis of CD4^+^ T Cells

Single-cell suspension of the intestine was prepared as described above and was used for the subsequent Fluorescence-activated cell sorting (FACS) analysis. The spleen samples were ground, filtered, and then suspended in 1 mL pre-cooled PBS (1×). The erythrocytes were lysed with lysis buffer, and the remained cells were washed and resuspended in FACS buffer (Cat#: 420201, Biolegend). Detailed procedure for FACS analysis was described in the supplementary material. Fluorescent-dye-conjugated antibodies used in this study are listed in **Table S5**. Relevant negative control, Fluorescence Minus One (FMO) control and each fluorescence compensation sample were used to adjust fluorescence compensation and identify the populations of interest. Cells were sorted on a BECKMAN COULTER flow cytometer (CytoFLEX S), and data were analyzed using FlowJo software (10.4.0).

### Statistical Analysis

SPSS 26.0 was used for statistical difference analysis of the experimental data and an unpaired two-tailed Student’s test was applied for two-group comparisons. All data with error bar was expressed as mean ± SEM. GraphPad Prism 9.0 software was used for plotting. Nominal *p* values of the results were corrected several times using the Benjamini-Hochberg method, and *p* values below 0.05 after correction were considered statistically significant.

## Results

### Intestinal Bacterial Perturbation Plays Critical Roles in HUA and Gout

*Uox*-KO mouse model, which spontaneously developed pronounced HUA and urate nephropathy and has been widely used in HUA and associated disorders (2), was used to assess the role of gut microbiota alteration in the pathogenesis of HUA and gout. Consistent with previous reports, we observed lower body weight (**Figure S1A**, *p*<0.01) and significantly elevated levels of SUA and UUA (**Figures S1B, C**, *p*<0.001) in *Uox*-KO mice. Inflammatory cell infiltration was observed in the *Uox*-KO mice footpad (**Figure S1D**). Animals that received monosodium urate (MSU) crystals showed increased footpad swelling and reduced mechanical pain threshold (**Figures S1E**–**G**, *p*<0.001 and *p*<0.01). Furthermore, reduced renal tubules, glomerular atrophy and peripheral fibrosis were observed (**Figure S1H**). Kidney index (**Figure S1I**, *p*<0.05), UCr, U-TP, and U-Alb (**Figures S1J**–**L**, *p*<0.001) were significantly reduced while SCr was markedly raised (**Figure S1M**, *p*<0.01). The *Uox*-KO mice have stable elevated SUA levels and mimicking the symptoms of gout patients. It is a suitable model to investigate the underlying mechanism of HUA and gout.

**Figure 1:**
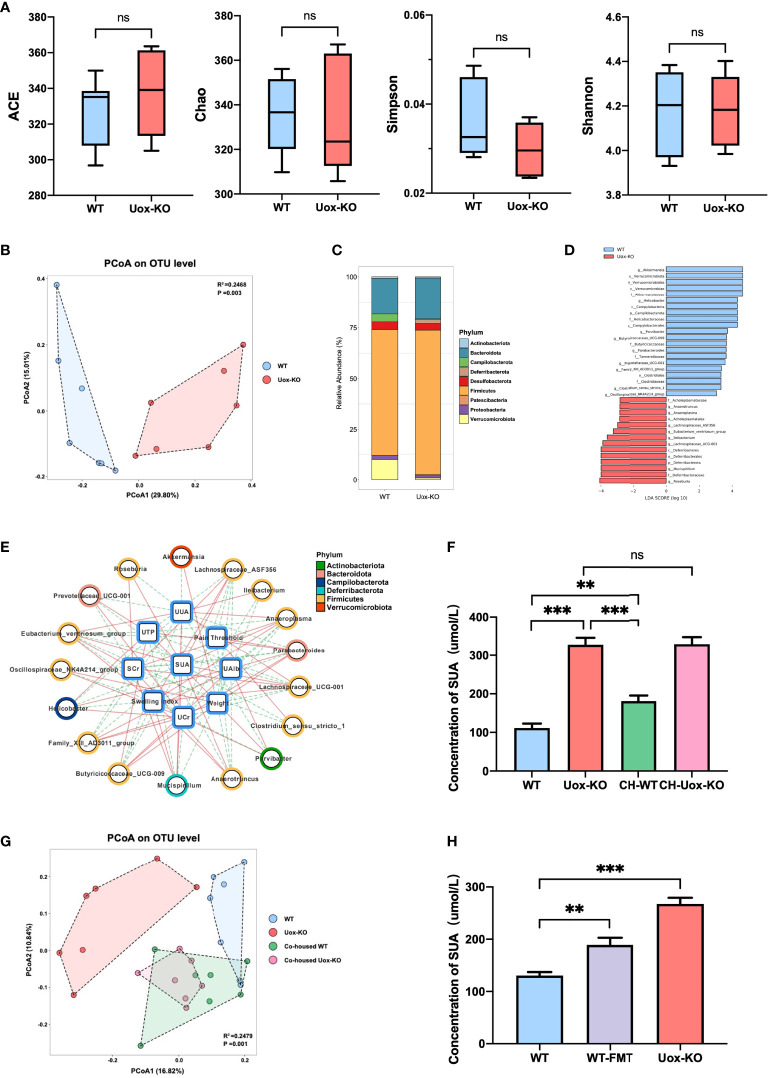
Changes in the gut microbiota composition with HUA and gout. **(A)** Species richness indices (ACE & Chao) and species diversity indices (Shannon & Simpson) were used to evaluate alpha diversity. **(B)** Principal coordinate analysis plot of beta diversity as measured by Bray-Curtis distance (*p*=0.003). **(C)** Mean relative abundance of taxa at phylum level. Legend lists only the top taxa. **(D)** A histogram of the LDA scores was computed for features that showed differential abundance between WT and *Uox*-KO mice. Only taxons with LDA scores >2 and *p <*0.05 are represented. **(E)** The correlation networks among disease indices and differential microorganisms. The line colors indicate positive correlation (red implementation) or negative correlation (green dashed line) (*p* < 0.05, r > 0.5 or r < - 0.5). **(F)** SUA concentration of co-housed mice. **(G)** PCoA analysis of co-housed mice (*p* =0.001). Co-housed WT had the similar composition of the gut microbiota with *Uox-*KO mice. **(H)** SUA concentration of WT-FMT mice. Values are expressed as mean ± SEM. “ns” represents not significant; **p < 0.01; ***p < 0.001.

To investigate if the gut microbiota plays any role in HUA and gout, we then compared the gut microbiota structure of *Uox*-KO mice and WT mice using 16S rRNA gene amplicon sequencing. Although there were no apparent differences in richness indices (Ace and Chao) or diversity indices (Simpson and Shannon) between *Uox*-KO mice and WT mice (**Figure 1A**), PCoA analysis from the OTU level revealed an apparent shift in the gut microbiota composition in *Uox*-KO mice (**Figure 1B**, p=0.003). We noted an expansion of Firmicutes (phylum), as well as a reduction of Verrucomicrobiota (phylum) relative abundance in *Uox*-KO mice compared to that in WT mice (**Figure 1C**). LefSe analysis demonstrated the genera *Akkermansia*, *Butyricicoccaceae_*UCG-00*9, Ruminococcus, Helicobacter* were overrepresented in WT mice. In contrast, the genera *Anaerotruncus*, *Anaeroplasma*, *Lachnospiraceae*_ASF356, *Eubacterium*_*ventriosum*, *Ileibacterium*, *Lachnospiraceae*_UCG-001, *Mucispirillum*, and *Roseburia* were enriched in *Uox*-KO mice, the majority of which belong to the Firmicutes phylum (**Figure 1D**). Spearman correlation analysis further demonstrated that the abundance of *Akkermansia* and *Butyricicoccaceae_*UCG-009, the strains overexpressed in WT mice, were positively correlated to body weight but negatively correlated to SUA (**Figure 1E**, *p* < 0.05, r > 0.5 or r < − 0.5). In contrast, the bacteria enriched in *Uox*-KO mice, particularly those from Firmicutes, were positively correlated with SUA and SCr, but negatively correlated with UTP, U-Alb and weight, implying the contribution of these bacteria to the pathogenesis of HUA and gout (**Figure 1E**).

We further confirmed these findings by a co-housing experiment, in which WT mice were co-housed with *Uox*-KO mice for 8 weeks. The co-housed WT mice displayed an increased SUA compared to that of the control WT mice (**Figure 1F**, *p*<0.01, *p*<0.001), while the co-housed *Uox*-KO mice still maintained the elevated level of SUA. 16S rRNA amplicon analysis further confirmed the transfer of the colonic microbiota from *Uox*-KO mice to the co-housed WT animals (**Figure 1G**, *p*=0.001). The abundance of the strains that positively correlated to SUA, such as *Ileibacterium*, *Anaerotruncus* and *Roseburia* was significantly increased in co-housed WT mice, but remained the similar abundance in co-housed KO mice (**Figure S2A**, *p*<0.01, *p*<0.05). In addition, to fully verify the effect of intestinal bacterial perturbation on HUA, we transplanted fecal microbiota of *Uox*-KO mice into WT mice by gavage for 4 weeks. In keeping with the co-housing experiment, WT-FMT mice showed a significantly increased SUA compared to normal WT mice (**Figure 1H**, *p*<0.01). WT-FMT mice possessed similar biological characteristics with *Uox*-KO mice, showing significantly increased UUA (**Figure S2B**, p<0.01) and markedly raised SCr (**Figure S2C**, p<0.05). Although no significant differences were observed in kidney index, UCr, U-TP, and U-Alb between normal WT and WT-FMT mice (**Figures S2D–G**), a decreasing tendency was detected in WT-FMT mice. The lack of significance may be due to insufficient FMT duration to cause kidney damage. These results fully certified that gut microbiota play an important role in hyperuricemia and gout. The long-term exposure to the specific bacteria from *Uox*-KO mice will cause the elevation of SUA.

### The Gut Microbiota Affects Host Amino Acid Metabolism in *Uox*-KO Mice

**Figure 2:**
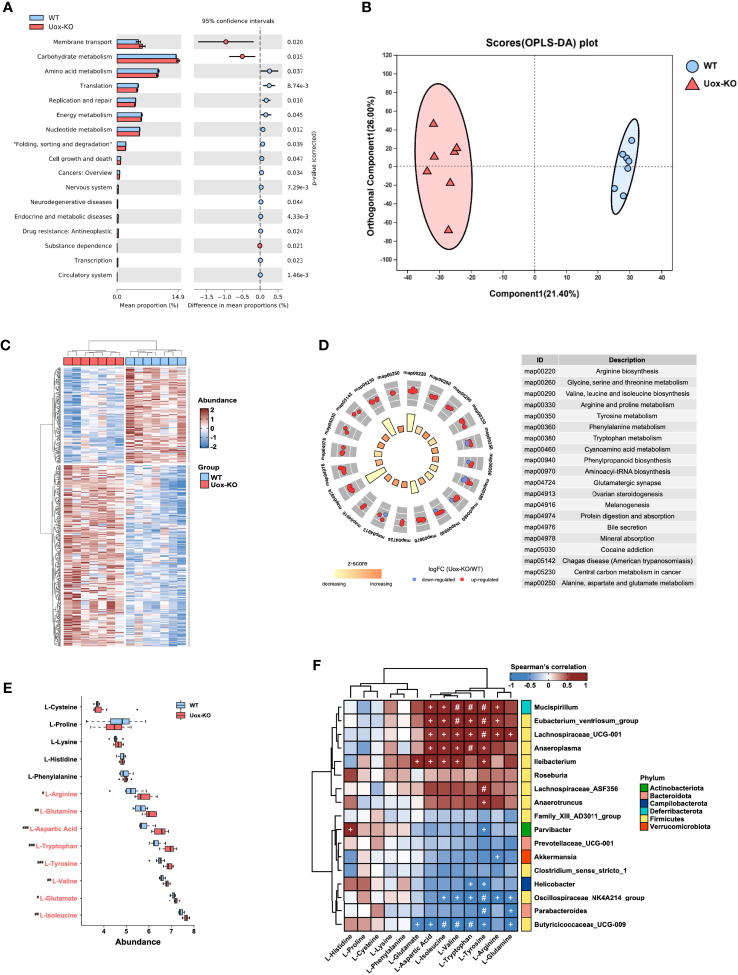
Gut microbiota affects host amino acid metabolism. **(A)** The functional potential of the bacterial communities on the basis of 16S rDNA sequencing profiles was predicted by PICRUSt2. The main metabolic functions involved include membrane transport, carbohydrate metabolism and amino acid metabolism. **(B)** The OPLS-DA score plot (the circle represents samples with 95% confidence interval). **(C)** The heatmap presentation of differential metabolites. **(D)** The top twenty enriched KEGG pathways of differentially expressed metabolites. **(E)** The relative abundance of fecal amino acids in WT mice and *Uox*-KO mice (N = 7 mice/group). Blue and red denote WT-enriched and *Uox*-KO enriched amino acids, respectively. Values are expressed as mean ± SEM. # denotes amino acids differed in abundance between WT and *Uox*-KO samples. ^#^ *p* < 0.05; ^##^ *p* < 0.01; ^###^ *p* < 0.001. **(F)** Spearman’s rank correlation analysis was conducted between discrepant microbial taxa and fecal amino acids. Positive correlations are displayed in red and negative correlations in blue. The intensity of the color is proportional to the correlation coefficient. ^+^ *p* < 0.05; ^#^ *p* < 0.01.

PICRUSt2 software was utilized to predict the functional gene compositions of gut microbiota based on 16S rRNA sequencing data. The results showed that most functional predicted categories are related to metabolic and cellular processes, which mainly involved in membrane transport, carbohydrate metabolism, and AA metabolism (**Figure 2A**). To further unveil the linkage between gut bacterial changes and metabolism alterations, we compared the fecal metabolic profiles of the two groups by untargeted LC-MS. As shown in **Figure 2B**, we observed a clear overall separation between two groups in the OPLS-DA analysis, and 444 differential fecal metabolites were found with VIP>1 (variable important in projection, VIP) and *p* < 0.05 (**Figure 2C**). In line with the predicted results by PICRUSt2, the top twenty enriched pathways obtained in KEGG pathway analysis were mainly involved in AA biosynthesis and metabolism (**Figure 2D**). Deeper analysis disclosed that the relative abundance of eight fecal AAs, including isoleucine (*p*<0.01), tryptophan (*p*<0.001), valine (*p*<0.01), arginine (*p*<0.05), glutamine (*p*<0.01), tyrosine (*p*<0.001), glutamate (*p*<0.05), and aspartic acid (*p*<0.001), were significantly accumulated in *Uox*-KO mice (**Figure 2E**). Among these eight differentially expressed AAs, five of them are essential- or conditional essential- AAs, which cannot be synthesized endogenously and depend largely on the fermentation of gut microbiota. It is worthy to note that the abundance of most AAs, which increased in feces, was statistically decreased in serum (**Figure S3A**). On the other hand, we also checked the metabolic profile in kidney. We found no statistically significant differences in these renal AAs between the two groups (**Figure S3B**), suggesting the changed AA serum concentration in *Uox*-KO mice was most likely caused by the intestinal disorders of AA metabolism rather than renal metabolism.

To better interpret the correlation between fecal metabolome and microbiome changes, we conducted the correlation analysis of the discrepant bacterial genera and fecal AAs. There was a strong positive correlation between those altered bacteria, such as *Mucispirillum*, *Eubacterium_ventriosum*, *Lachnospiraceae*_ UCG_001, *Anaeroplasma*, and *Ileibacterium*, and the changed fecal AAs, but has no significant correlation with unchanged AAs (**Figure 2F**). In addition, we also observed a significant correlation between gut microbiota and serum levels of AA (**Figure S3C**), and therefore speculated that the perturbation of gut microbiota contributes to altered serum AA metabolome. No obvious relationship between gut microbiota and renal levels of AA was detected (**Figure S3D**).

### Disordered Amino Acid Metabolism Correlated With Disease and Along With Impaired SLCs Expression

**Figure 3:**
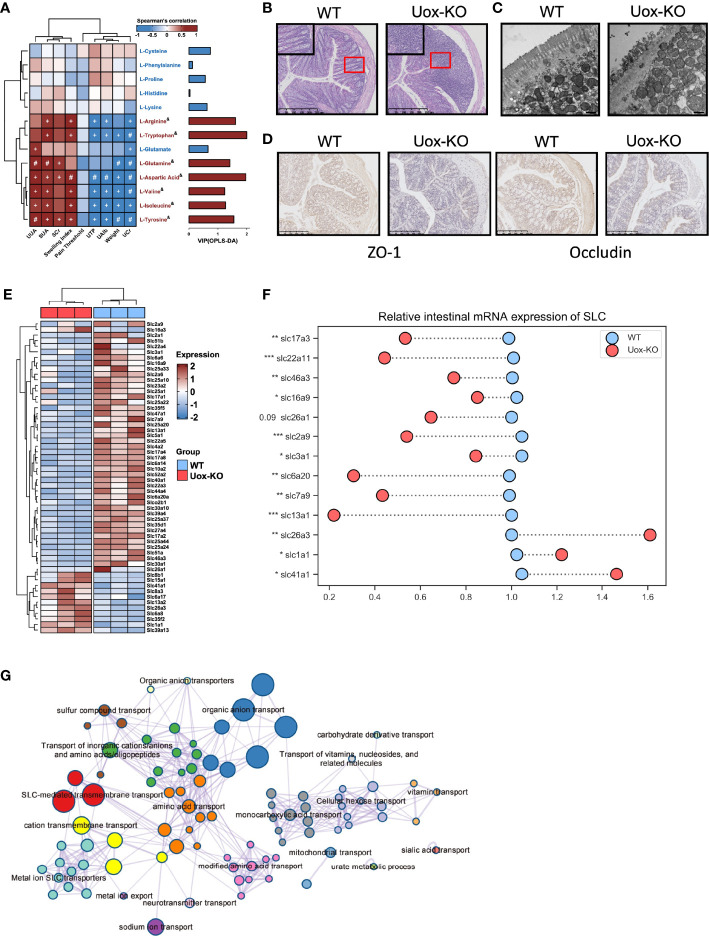
Impaired intestinal mucosal barrier in *Uox*-KO mice. **(A)** Analysis of Spearman’s rank correlation between 13 feces amino acids and gout symptoms. Positive correlations are displayed in red and negative correlations in blue. The intensity of the color is proportional to the correlation coefficient. ^+^ *p* < 0.05; ^#^ *p* < 0.01; & on behalf of VIP>1. **(B)** H&E-stained of colonic sections (Scale bar: 250 µm). The boxed areas are shown enlarged. **(C)** Transmission electron micrograph of the intestinal villa in colon of WT and *Uox*-KO mice (Scale bar: 500 nm). **(D)** Expression of ZO-1 and Occludin in the colon was measured by immunohistochemistry (Scale bar: 250 µm). **(E)** Heatmap of 56 differentially expressed SLC genes in colon between WT and *Uox*-KO mice based on RNA transcriptome sequencing. **(F)** *q*RT-PCR validation of mRNA expression of randomly chosen SLC genes in mice intestinal tissues. **(G)** Network of enriched terms: colored by cluster ID, where nodes that share the same cluster ID are typically close to each other; colored by *p*-value, where terms containing more genes tend to have a more significant *p*-value. Values are expressed as mean ± SEM. *p < 0.05; **p < 0.01; ***p < 0.001.

AAs involve in the various biochemical processes including the biosynthesis of UA, and previous studies have demonstrated the associations of plasma AAs and HUA and gout (18). However, the investigation is limited on the contributions of disordered fecal AA to HUA and gout. We performed a series of Spearman correlation studies to elucidate the relationship between fecal AAs and disease indices. As shown in **Figure 3A**, most of the altered fecal AAs were positively correlated with SUA, UUA, SCr, and footpad swelling degree in *Uox*-KO mice, while inversely correlated with body weight, UCr, U-TP and U-Alb. These results suggested the importance of impaired fecal AA metabolism in the pathogenesis of HUA and gout. Although no correlation with mechanical pain threshold was found in our study, it can be explained by the short time for measuring mechanical pain threshold after MSU injection.

Since the integrity of the intestinal barrier is essential for the absorption of nutrients and its dysfunction has been implicated in numerous gastrointestinal and non-gastrointestinal diseases (19), we even checked the integrity and inflammatory status of the intestine. In line with our expectation, massive infiltration of inflammatory cells (**Figure 3B**), severe atrophy of the intestinal villi (**Figure 3C**), and loss of the tight junction proteins ZO-1 and Occludin (**Figure 3D**) were observed in *Uox*-KO mice. We next compared the gene expression profiling in the intestine by RNA transcriptome sequencing. We mainly focused on the alteration of the SLCs (Solute carrier, the membrane transporters for AAs transportation) family expression. Whole transcriptional analysis revealed profound alteration in the SLC family profile. Fifty-six SLC transporters displayed different expression patterns. Most of them were downregulated in *Uox*-KO mice (**Figure 3E**). Changes were confirmed by *q*PCR in a randomly chosen subset of SLCs (**Figure 3F**, *p*<0.05, *p*<0.01, *p*<0.001). What’s more, the expression of SLC22A11 and SLC17A3, the confirmed renal urate transporter, were also checked by *q*PCR (**Figure 3F**, *p*<0.05, *p*<0.01, *p*<0.001). Further pathway and network enrichment analyses of the differentially expressed SLCs showed that these SLC transporters belong to 20 pathways. Amino acid transport and modified amino acid transport pathways were significantly enriched, which is consistent with the results of metabolomics. In addition, urate metabolic process was also activated, indicating some unspecified SLCs were involved in the uric acid metabolism as well (**Figure 3G** and **Table S6**).

### Amino-Acid Metabolism Disorders Perturb Intestinal T-Cell Homeostasis

**Figure 4:**
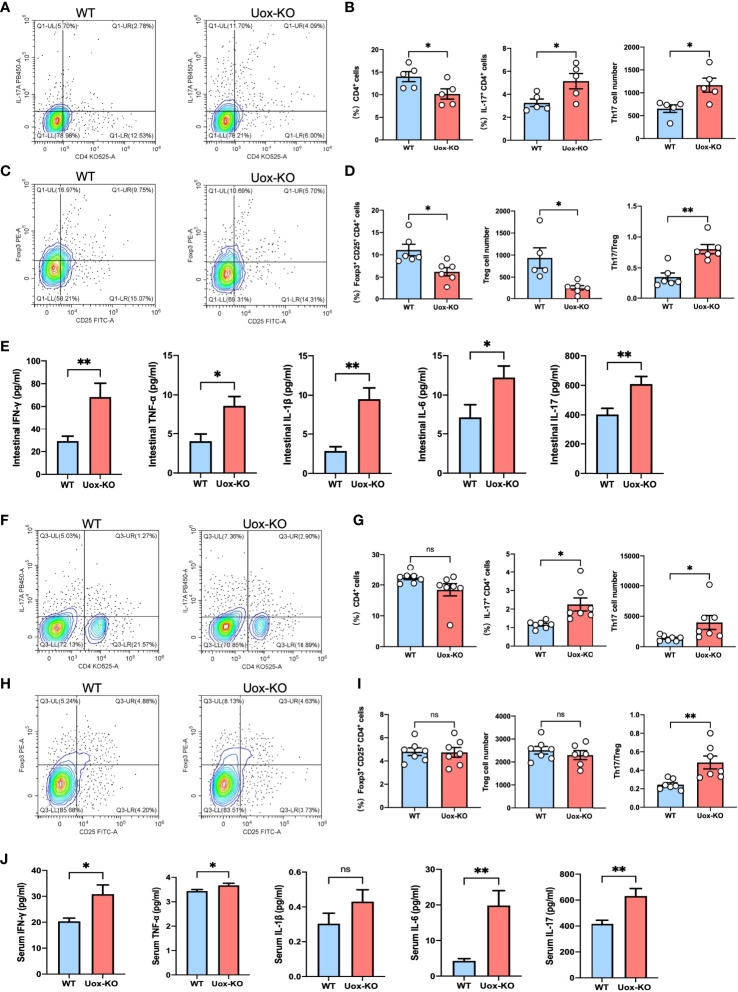
Disruption of intestinal CD4^+^ T-cell homeostasis in *Uox*-KO mice. **(A)** Representative flow cytometry plots of the intestinal Th17 populations, which were measured as IL-17a^+^ CD4^+^ CD45^+^ live cell percentages. **(B)** Percentage of intestinal CD4^+^ T cells, percentage and absolute number of intestinal Th17 cells, showing decreased population of CD4^+^ T cells and increased differentiation of Th17 cells in *Uox*-KO mice. **(C)** Representative flow cytometry plots of the intestinal Treg populations, which were measured as Foxp3^+^ CD25^+^ CD4^+^ CD45^+^ live cell populations. **(D)** Percentage and absolute number of intestinal Treg cells and the ratio of Th17/Treg cells, showing decreased Treg cells and increased Th17/Treg ration in *Uox*-KO mice. **(E)** The levels of intestinal IFN-γ, TNF-α, IL-1β, IL-6 and IL-17 were measured by ELISA. **(F)** Representative flow cytometry plots of the spleen Th17 populations. **(G)** Percentage of spleen CD4^+^ T cells, percentage and absolute number of Th17 cells. **(H)** Representative flow cytometry plots of the spleen Treg populations. **(I)** Percentage and absolute number of spleen Treg cells and the ratio of Th17/Treg cells in spleen. **(J)** The levels of serum IFN-γ, TNF-α, IL-1β, IL-6 and IL-17. Values are expressed as mean ± SEM. “ns” represents not significant; *p < 0.05; **p < 0.01.

It is well known that AAs are vital nutrients for T cells, and efficient transportation of exogenous AAs is the prerequisite for T-cell activation, differentiation, and function (20). We, therefore, hypothesized that the observed AAs disorders might be associated with T cell dysfunction. To test this hypothesis, we evaluated the proliferation of CD4^+^ T cells in the intestine of *Uox*-KO mice by FACS. It was subsequently found that the intestinal CD4^+^ T cell counts were quantitatively reduced and the percentage and absolute number of pro-inflammatory CD4^+^ IL-17^+^ (Th17) cells were increased in *Uox*-KO mice (**Figures 4A, B**, *p*<0.05), along with a decrease in the percentage and absolute number of CD4^+^ CD25^+^ Foxp3^+^ (Treg) cells and a significant increase in Th17/Treg ratio (**Figures 4C, D**, *p*<0.05, *p*<0.01). Consistent with the results of flow cytometry, ELISA analysis revealed the heightened levels of IFN-γ (*p*<0.01), TNF-α (*p*<0.05), IL-1β (*p*<0.01), IL-6 (*p*<0.05), and IL-17 (*p*<0.01) in intestinal homogenates of *Uox*-KO mice (**Figure 4E**). Similar phenotypes were also observed in the spleen of *Uox*-KO mice (**Figures 4F**–**J**).

Besides, the mantel test results indicated that the count of CD4^+^ T cells was significantly correlated with featured bacteria, differential fecal AAs, and disease indices (**Figure S4A**). These results suggested that gut microbiome perturbation plays a vital role in AA metabolism, which may affect the activation and differentiation of CD4^+^ T cells, and ultimately influence the occurrence and development of HUA and gout.

### Depletion of Amino Acids Blunts CD4^+^ T Cells *via* AKT-mTOR Pathway

**Figure 5:**
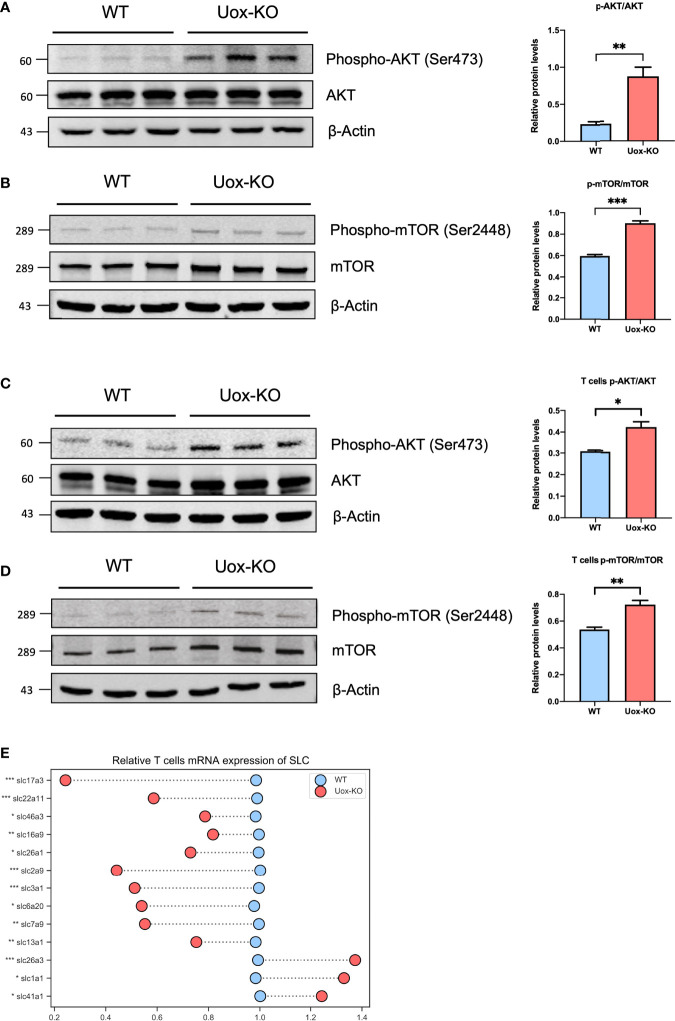
Activation of the Akt-mTOR pathway in intestine of *Uox*-KO mice. **(A)** The expression of total Akt and phosphorylation at the Ser473 residue in the colon were evaluated by Western blotting analysis. The gray value ratios of the phosphorylated Akt bands over the internal reference β-actin band were used to quantify the expression of phosphorylated Akt by Image J software. **(B)** The expression of total mTOR and phosphorylation at the Ser2448 residue in the colon were evaluated as stated above. **(C)** The expression of total Akt and phosphorylation at the Ser473 residue in the intestinal CD4^+^ T cells. **(D)** The expression of total mTOR and phosphorylation at the Ser2448 residue in the intestinal CD4^+^ T cells. **(E)** mRNA expression of SLC transporters in CD4^+^ T cells. Values are expressed as mean ± SEM (N = 3 mice/group). *p < 0.05; **p < 0.01; ***p < 0.001.

Several studies have reported that the influence of AA metabolism on T-cell fate decisions may largely depend on the mechanistic of rapamycin complex 1 (mTORC1) signaling (21). To this end, we adopted western blotting analysis and found the phosphorylation levels of AKT (Ser473) and mTOR (Ser2448) were dramatically increased in the intestine of *Uox*-KO mice (**Figures 5A, B**, *p*<0.01, *p*<0.001). We further isolated CD4^+^ T cells from the gut and verified this mechanistic pathway at the cell level (**Figures 5C, D**, *p*<0.05, *p*<0.01). In addition, to further clarify the role of amino acid deprivation in AKT activation, we also quantified the expression of SLCs in the isolated T cells by *q*PCR and observed a reduction in SLCs expression as well (**Figure 5E**, *p*<0.05, *p*<0.01, *p*<0.001), which is consistent with the results of the whole intestine. Taken together, these results suggested that the unavailability of amino acids caused by SLC transporters dysfunction led to the disorder of T cell proliferation and differentiation *via* the AKT-mTOR pathway, and as a consequence, the aberrant Th17 result in the intestinal inflammation in *Uox*-KO mice.

## Discussion

**Figure 6:**
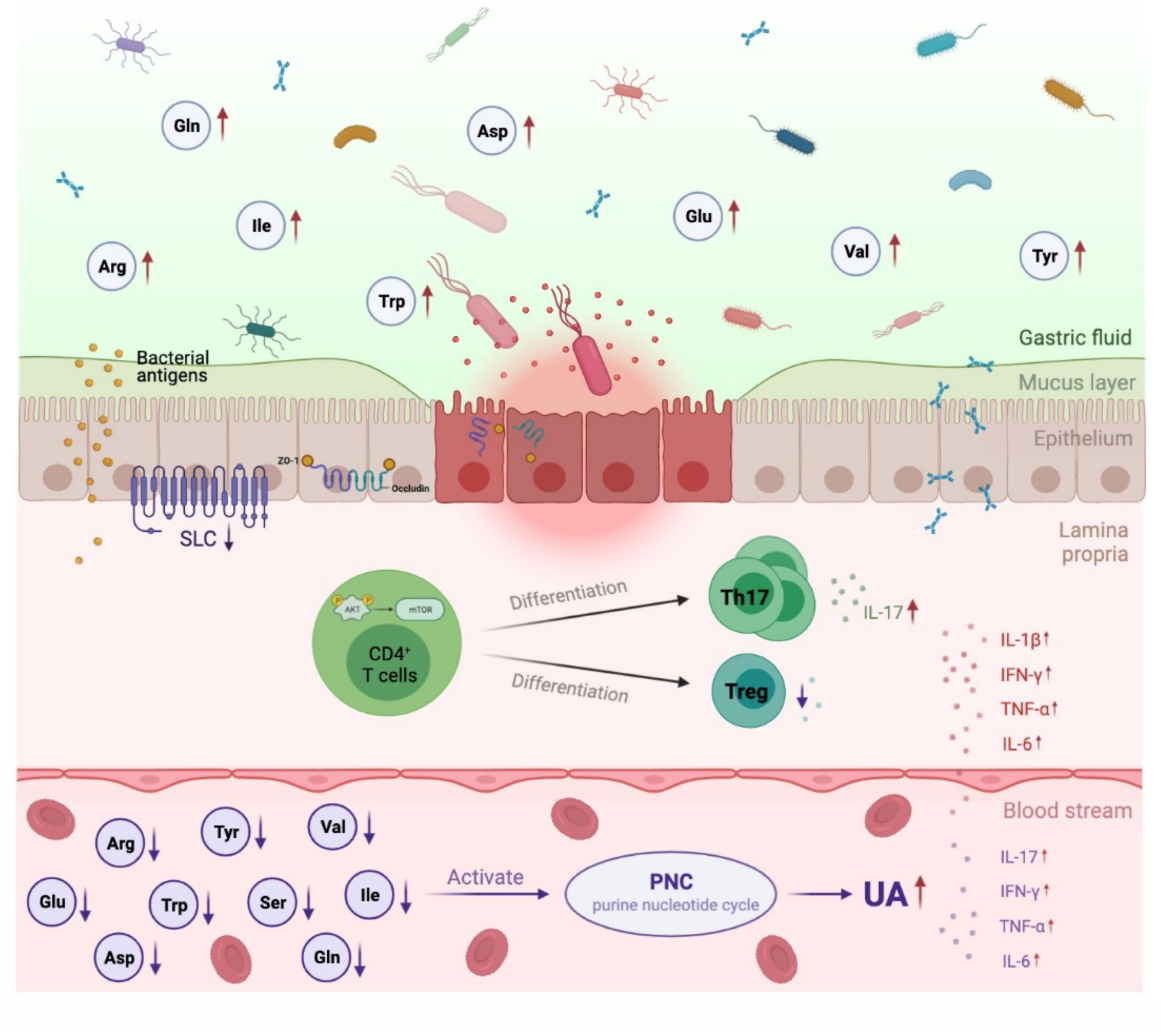
Proposed mechanism of perturbed gut microbiome in pathogenesis of HUA and gout. In *Uox*-KO mice, altered intestinal microflora composition and function resulted in the impairment of intestinal integrity and the disorder of SLCs family expression, which subsequently led to the dysfunction of AA transportation and the decreased serum levels of valine, isoleucine, glutamine, arginine, tryptophan and tyrosine. Deficiency of serum amino acids further influenced purine nucleotide cycle and differentiation of CD4^+^ Th17, and eventually contributed to the occurrence and development of HUA and gout. Red arrow indicates upregulation, purple arrow indicates downregulation.

Cumulative evidence has demonstrated a great role for the gut microbiota and their metabolites in the pathogenesis of HUA and gout (22). However, the pathophysiologic mechanism of microbiota alterations in HUA and gout is not fully understood. In the current study, we proposed a conceptual advancement in understanding the mechanism of HUA and gout pathogenesis and revealed that dysbiosis of the gut microbiota may affect to the dysfunction of AA transportation and metabolism, which further promoted purine nucleotide cycle and Th17 cell infiltration (**Figure 6**).

Previous studies have suggested the direct or indirect involvement of bacterial metabolites, particularly short-chain fatty acids (SCFAs) and bile acids (BAs), in inflammatory disease. In the present study, we detected many fecal metabolites significantly altered in *Uox*-KO mice compared to WT mice. Among them, the most significant change occurred in AAs, particularly those in branched-chain AAs (BCAAs, valine, and isoleucine), α-ketoglutarate family (glutamate, glutamine, and arginine), and aromatic AAs (AAAs, tryptophan and tyrosine). The mutual interplay between AAs and gut bacteria has been extensively discussed (16, 23–25). For example, as the most abundant essential AAs, the levels of BCAAs are tightly correlated with certain specific bacteria such as *Akkermansia* (26, 27), which significantly depleted in *Uox*-KO mice.

A very recent study reported that HUA and gout patients have distinct serum metabolomics signatures, and arginine metabolism appears to play a critical role (28). In our study, we discovered a panel of AAs were strongly related to HUA and gout in addition to arginine. For instance, BCAAs deficiency leads to oxidative stress and major increases in the metabolites of the purine nucleotide cycle (PNC). The PNC acts as a temporary purine nucleotide reservoir and is activated during high ATP utilization or impaired oxidative phosphorylation. Catabolism of the purine nucleotides ultimately leads to the uric acid production, suggesting the lower plasma BCAAs arouse the higher serum uric acid (29, 30). Additionally, glutamine is a major metabolite of the TCA cycle in energy metabolism and a precursor of urate synthesis. A defect of glutamine metabolism maybe was caused by accelerated purine production (28). What’s more, the disorder of aromatic amino acids metabolism is one of the leading causes of gout (31). It has been reported that the administration of tryptophan suppressed the elevation in SUA levels and increased urinary UA excretion by regulating the metabolism of glycine to creatinine. The low serum tryptophan level maybe is an indicator of elevated UA synthesis (32). And last, tyrosine catalyzes the formation of deoxyribose of pyrimidine and purine ribonucleotides (33, 34).

In addition to the effect on purine metabolism and UA generation, AA auxotrophy has evolved to become an immunoregulatory control point. The deficiency of AAs has long been to impair immune function. It is the signal that initiates the complete signaling response, which increase the susceptibility to many major infectious and inflammatory diseases (35). The activation, differentiation and function of T cells heavily rely on environmental AAs (36, 37). Although it is widely accepted that gout is an inflammatory disease characterized by urate crystal-induced NLRP3 inflammasome activation with up-regulated caspase-1 protease and IL-1β in macrophages, recent evidence has highlighted that serum IL-17 levels are significantly elevated in gout patients (38). IL-17 is an essential pro-inflammatory cytokine and is mainly secreted by the CD4^+^ Th 17 cells and subsets of innate lymphoid cells (39–41). Many studies have shown that Th17/Treg balance in intestinal mucosa plays a fundamental role in stabilizing gut immunological homeostasis and a shift Th17/Treg equilibrium towards the pro-inflammatory Th17 side is involved in the occurrence and development of several autoimmune disorders (41–44). The same phenomenon was observed in our *Uox*-KO mice, suggesting a pathogenic role of this specific CD4^+^ T cell subset in gout occurrence and development (45). In fact, an aspect that should not be omitted is that the presence of IL-17. IL-17 has been proposed to be the main contributor to chronic inflammation at the later stages of an inflammatory response due to its ability to sustain the recruitment of neutrophils and inflammatory monocytes. It also can amplify the inflammation induced by other cytokines, including IL-1, IL-6, IL-8, and TNF-α (46, 47). Several studies have reported that serum or urinary levels of IL-17 are significantly elevated in patients with rheumatoid arthritis, multiple sclerosis, systemic lupus erythematosus, and autoimmune hepatitis (48–51). In the current study, the increased intestinal and serum levels of IL-17 were observed, along with the increase of IL-6, and TNF-α. We therefore hypothesized imbalanced Th17, and IL-17 are involved in the pathogenesis of gouty inflammation (38).

It is well-established that the intestinal barrier is ingeniously modulated by gut microbiota and host immune cells (52). Microbiota-derived metabolites, including SCFAs, AAs and BAs, are key mucosal barrier modulators (53–57). Disturbance of intestinal mucosal barrier function has been implicated in the pathogenesis and development of several disease states, including food allergies, inflammatory bowel disease, celiac disease, irritable bowel syndrome, metabolic syndrome, diabetes, non-alcoholic fatty liver disease and septic shock (58). Lv et al. (59) have reported that the altered microbiota composition in *Uox*-KO mice result in gut immune disorders and intestinal barrier dysfunction by upregulating TLR2/4/5 and promoting the release of IL-1β and TNF-α, which eventually promote the inflow of microorganisms in systemic circulation and lead to systemic inflammation. In our study, we observed the dysregulated intestinal immunity and compromised intestinal barrier as well, and demonstrated that damaged intestinal integrity contributes to the pathogenesis of HUA. However, we proposed a mechanistic understanding that perturbations in the gut microbiota is involved in the occurrence and progression of disease through dysfunction of AA transportation and metabolism, which further promoted purine nucleotide cycle and Th17 cell infiltration.

What’s more, mucosae are also responsible for AA transportation across the plasma membrane, which is mainly mediated by the SLC transporters. SLC transporters are an important class of membrane proteins and mediate many essential physiological functions including nutrient uptake, ion influx/efflux and waste disposal. Among them, SLC22A11, SLC17A3 and SLC2A9 have been identified as renal urate transporter that influences serum urate concentration and urate excretion (34, 60–62). Our results suggested that suppressing of these urate transporters in the intestine might impact extra-renal urate excretion and thereby influence SUA level. Other transporters, such as SLC16A9, SLC17A4, and SLC17A1, are also known to be involved in the UA regulation (62). Here, we revealed the profound alteration in the SLC family profile in *Uox*-KO mice intestine and intestinal T cells. Many of them have not been reported previously to be involved in HUA and gout. In line with these findings, a two-sample Mendelian randomization analysis based on the results from genome-wide association studies including >140,000 individuals of European ancestry revealed the contribution of abnormal AA and aberrant SLCs to gout (**Table S7**). These findings highlight the role of the abnormal AAs transportation and metabolism by gut microbiota in HUA and gout, and suggest that intestinal dysbiosis due to an impaired intestinal barrier may be the key cause of metabolic disorders in HUA mice.

Meanwhile, our study has several limitations. Though we have revealed the potential role of gut microbiota in the pathogenesis of HUA and gout, future studies to identify the key bacteria strains that may account for metabolites and inflammation will be critical. Beyond the AAs that have been studied in this study, further exploration will be required to understand other mechanistic links connecting gut microbiota and gout. Additional cell and faecal transplant studies are needed to confirm this fully. Future research into these critical questions may contribute to a better understanding of the pathogenesis of the disease and its broader potential clinical treatment.

## Data Availability Statement

The datasets presented in this study can be found in online repositories. The names of the repository/repositories and accession number(s) can be found in the article/**Supplementary Material**.

## Ethics Statement

The animal study was reviewed and approved by the Laboratory Animal Management and Welfare Ethical Review Committee of Zhejiang Chinese Medical University.

## Author Contributions

All authors were involved in drafting or revising the manuscript. TS and SS conceived and designed this study. SS, YL, YM, XW, MF, ZH, and YS were responsible for the acquisition and analysis of data. TS and SS drafted the manuscript. CW and TS approved the final version to be published. All authors contributed to the article and approved the submitted version.

## Funding

This work was supported by grants from the National Natural Science Foundation of China (81873145 & 82074248), the Natural Science Foundation of Zhejiang province (LY21H290005), and Graduate Scientific Research Fund Project of Zhejiang Chinese Medical University (2020YKJ09).

## Conflict of Interest

The authors declare that the research was conducted in the absence of any commercial or financial relationships that could be construed as a potential conflict of interest.

## Publisher’s Note

All claims expressed in this article are solely those of the authors and do not necessarily represent those of their affiliated organizations, or those of the publisher, the editors and the reviewers. Any product that may be evaluated in this article, or claim that may be made by its manufacturer, is not guaranteed or endorsed by the publisher.
