# Supplementary material for: Alteration of Gut Microbiome and Correlated Amino Acid Metabolism Contribute to Hyperuricemia and Th17-Driven Inflammation in Uox-KO Mice
Source: Front Immunol. 2022 Feb 7;13:804306. doi: 10.3389/fimmu.2022.804306 (PMC8858814; doi:10.3389/fimmu.2022.804306)
Supplement: Supplementary file 1 [file DataSheet_1.pdf]

Supplementary Material

Alteration of gut microbiome and correlated amino acid metabolism contribute to hyperuricemia and Th17-driven inflammation in *Uox*-KO mice

Siyue Song[1†], Yu Lou[1†], Yingying Mao[2], Xianghui Wen[1], Moqi Fan[1], Zhixing He[1], Yang Shen[3], Chengping Wen[1*], Tiejuan Shao[1*]

[1]College of Basic Medical Sciences, Zhejiang Chinese Medical University, Hangzhou, China.

[2]School of Public Health, Zhejiang Chinese Medical University, Hangzhou, China.

[3]The Second Clinical Medical College, Zhejiang Chinese Medical University, Hangzhou, China

† These authors have contributed equally to this work.

***Corresponding authors:** Prof. Chengping Wen, Email: wengcp@163.com

Or Prof. Tiejuan Shao, tiejuanshao@zcmu.edu.cn

Financial support: This research was supported by grants from the National Natural Science Foundation of China (81873145 & 82074248), the Natural Science Foundation of Zhejiang province (LY21H290005), and Graduate Scientific Research Fund Project of Zhejiang Chinese Medical University (2020YKJ09).

Running head: Altered microbiome in *Uox*-KO mice.

Competing interests: The authors declare that they have no conflict of interest.

Supplementary Table 1. Primers for mice genotyping

Primers	Sequence (5'→3')
P1	AATCAAGGCAAGAAAGGCAAGC
P2	CCTCAACAAATCAATGGGACACC
P3	GGCAGGTGTCTTTTGTTGATGTTTTG
P4	TCCCGTGAGTAGGCATTTGGTGA

Supplementary Table 2. Primary antibodies used for IHC analysis

Antibodies	Source	Cat#
ZO-1	Affinity Biosciences	#AF5145
Occludin	Affinity Biosciences	#DF7504

Supplementary Table 3. Primers for *q*RT-PCR analysis

Primers	Forward sequences	Reverse sequences
SLC22A11	GCTACCAGAATCGGCACGCT	CACCGGGAAGTCCACAATCC
SLC17A3	CATTGCCATGTAGCACAAAACA	CCCTGGTTCTATATTCAAAGATTGC
SLC6A20	CCCTGGTTCTATATTCAAAGATTGC	CACAGTTGACAAGGCACACC
SLC7A9	GCTCTTGCAGTCCCAGGCT	GGGACTACCCAAGATGCTGGA
SLC3A1	TGGAAGCGAAGGATCTGAGAA	GCTCTGAGTAGTGGGTGACCG
SLC1A1	TGTTGACTGGCTCCTGGACC	GCTCCAGCTCCTTCTTCGAGA
SLC41A1	GCCTCCCTGGTACTGGGTAT	GATTGGGATCAACCCAGACA
SLC2A9	ATCATTGTCCCTGCCTTGGTC	TGACCTGCCAGCGGACAAA
SLC13A1	CTATCGCCGCTTTCTCCTTGT	GGCAGTAGAGCTGTGATTGACA
SLC26A1	CAGAAGGGTGGGACACTGGTA	ACATCACCTGCTAGGTATTCCT
SLC26A3	AACATCCCTCCAGCCTACG	TGGACCCACAGATATGTGTCT
SLC16A9	CCGTTAGCTGTTGGGGTCTT	TGACGGGTCTTGCTCCAAAA
SLC46A3	GGAGGAAGTTCAGAAAAAGGCATC	TGTCGCTGCTCGCTAAAAGC
β-actin	GGCTGTATTCCCCTCCATCG	CCAGTTGGTAACAATGCCATGT

Supplementary Table 4. Primary antibodies used for western blotting analysis

**Antibodies**	**Dilution**	**Source**	**Cat#**
AKT	1:1000	CST	#4691
p-AKT (Ser473)	1:1000	CST	#4060
mTOR	1:1000	CST	#2972
p-mTOR (Ser2448)	1:1000	CST	#2971
β-actin	1:1000	CST	#4970

Supplementary Table 5. Antibodies used for flow cytometry analysis

**Antibodies**	**Fluorophore**	**Source**	**Cat#**
CD45	BV605	BD Pharmingen	#563053
CD4	BV510	BD Pharmingen	#563106
CD25	BB515	BD Pharmingen	#564424
FOXP3	PE	Thermo Fisher	#12-5773-82
IL-17A	BV421	BD Pharmingen	#563354
Fixable Viability Stain 700	APC700	BD Pharmingen	#564997

Supplementary Table 6. Amino Acid Transports and their Substrates

SLC	Substrates	References
SLC17A3	Total homocysteine	(1, 2)
SLC22A11	Glu, Asp	(3)
SLC46A3	-	-
SLC16A9	Monocarboxylic acids	(4, 5)
SLC26A1	Ala, Val	(6)
SLC2A9	Cys, Trp, Ile, Pro	(7, 8)
SLC3A1	Cys, Arg	(9, 10)
SLC6A20	Pro, Gln, Gly, hydroxyproline	(11)
SLC7A9	Cys, neutral amino acid	(9, 10)
SLC13A1	Ser, Thr	(12)
SLC26A3	Oxalate	(13)
SLC1A1	Glu, Asp, Cys	(14, 15)
SLC41A1	Magnesium transporters	(16)

Supplementary procedure for LC-MS analysis

The chromatographic separation was performed at 40 ℃ with a column of BEH C18 (100 mm × 2.1 mm, 1.7 µm, Waters, Milford, MA, USA). The two mobile phases consisted of 0.1 % formic acid aqueous solution (solvent A) and acetonitrile/ isopropanol (v/v, 1:1) with 0.1 % formic acid (solvent B). The flow rate was 0.40 mL/min and mobile phase (A: B) elution gradient was as follows: 95 %: 5 % for 0 min, 80%: 20% for 0 to 3.0 min, 5 %: 95 % for 3.0 to 9.0 min, 5 %: 95% for 9.0 to 13.0 min, 95 %: 5 % for 13.0 to 13.1 min, and 95 %: 5 % for 13.1 to 16.0 min. As a part of the system conditioning and quality control process, a pooled quality control sample (QC) was prepared by mixing equal volumes of all samples. The QC samples were disposed and tested in the same manner as the analytic samples.

Supplementary procedure for transcriptome analysis

The raw paired end reads were trimmed, and quality controlled by SeqPrep (https://github.com/jstjohn/SeqPrep) and Sickle (https://github.com/najoshi/sickle) with default parameters. The clean reads were separately aligned to reference genome with orientation mode using HISAT2 (http://ccb.jhu.edu/software/hisat2/index.shtml) software. The mapped reads of each sample were assembled by StringTie (https://ccb.jhu.edu/software/stringtie/index.shtml). The expression level of each transcript was normalized by FRKM method (Fragments per kilobof exon per million mapped reads).

Supplementary procedure for Western blotting analysis

Equal mass of intestinal proteins (30 µg/lane) were used for electrophoresis in a 10% or 8% SDS-PAGE gel. Protein bands in the gel of SDS-PAGE were electrophoretically transferred onto PVDF membrane. After blocking with 5 % milk in TBST buffer for 1 h at room temperature, the membranes were incubated with the primary antibodies overnight at 4 ℃ with shaking. The membranes were then washed 3 times with TBST and incubated with secondary antibody for 1 h at room temperature.

Supplementary procedure for Flow cytometry analysis

For cell surface staining, single-cell suspensions were washed and stained with fluorescence-conjugated antibodies for 30 minutes at room temperature, washed, and then resuspended in Cell Staining Buffer (Cat#: 420201, Biolegend). For intracellular cytokine staining, cells were restimulated in complete 1640 medium with a cell activation cocktail containing Brefeldin A (Cat#: 423304, Biolegend) for 6 h. Cells were fixed and permeabilized using the BD Cytofix/Cytoperm^{TM} buffer system. Cells were stained with the following fluorescent antibodies: CD45 BV605, CD4 BV510, CD25 BB515, FOXP3 PE, IL-17A BV421. We gated on live CD45$^+$ cells, CD4$^+$ cells and IL-17A$^+$ cells. CD4$^+$/IL-17A$^+$ double-positive cells were used to determine the percentages of Th17 cells. We gated on live CD45$^+$ cells, CD4$^+$ cells, CD25$^+$ cells and Foxp3$^+$ cells. CD25$^+$/Foxp3$^+$ double-positive cells were used to determine the percentages of Treg cells.

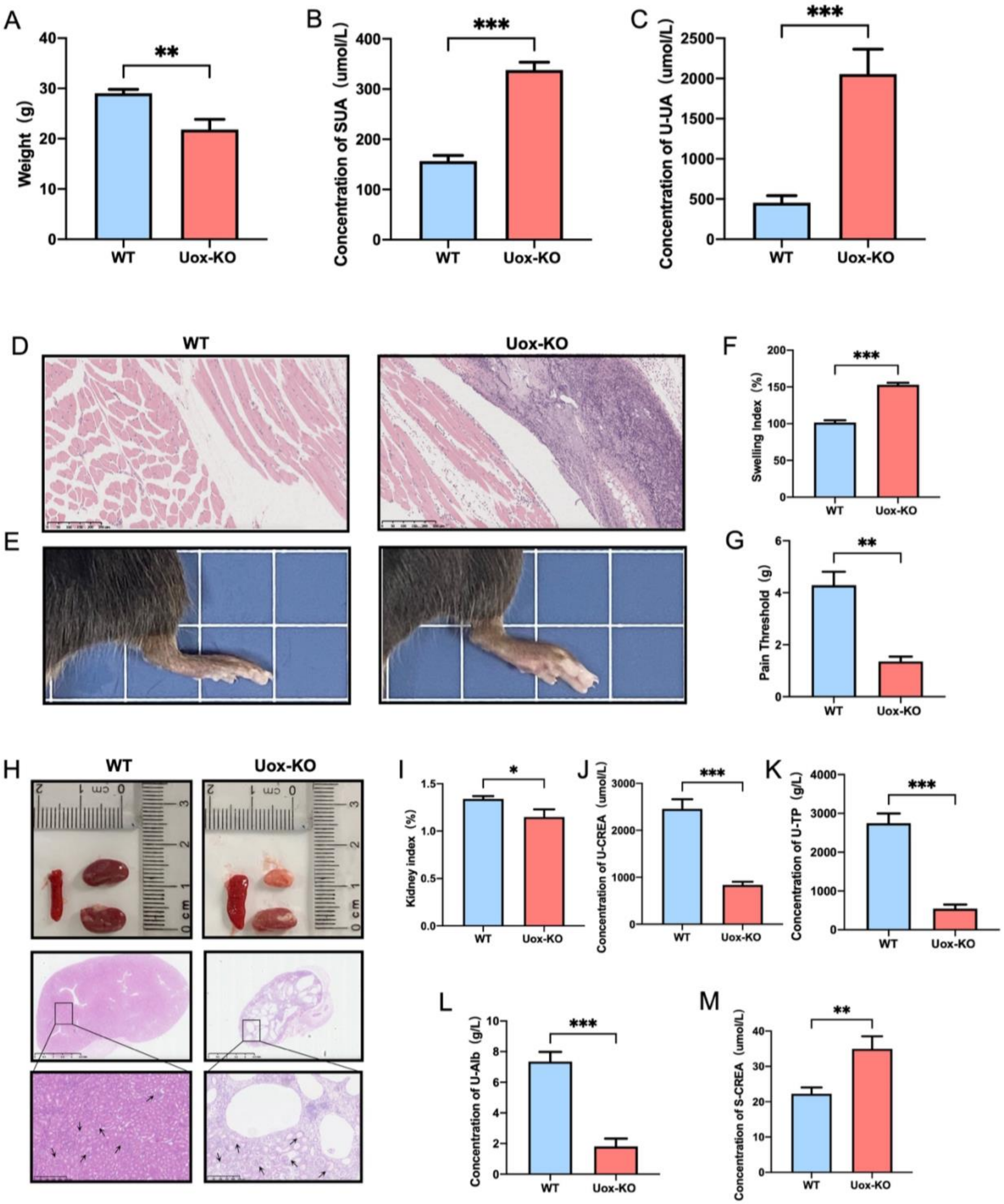

Figure S1

(A) Body weight of WT and *Uox*-KO mice. **(B)** The level of SUA. **(C)** The level of UUA. **(D)** Hematoxylin and eosin staining of mice claw. **(E-F)** Footpad swelling. **(G)** Mechanical pain

threshold. **(H)** Hematoxylin and eosin staining of whole-kidney longitudinal sections, black arrows indicate renal tubules, collapsed and necrotic nephrons. **(I)** Kidney index. **(J)** The concentration of urinary creatinine. **(K)** The concentration of urinary total protein. **(L)** The concentration of urinary albumin. **(M)** The level of serum creatinine. (N = 7 mice/group). Values are represented as mean ± SEM. * $p < 0.05$; ** $p < 0.01$; *** $p < 0.001$.

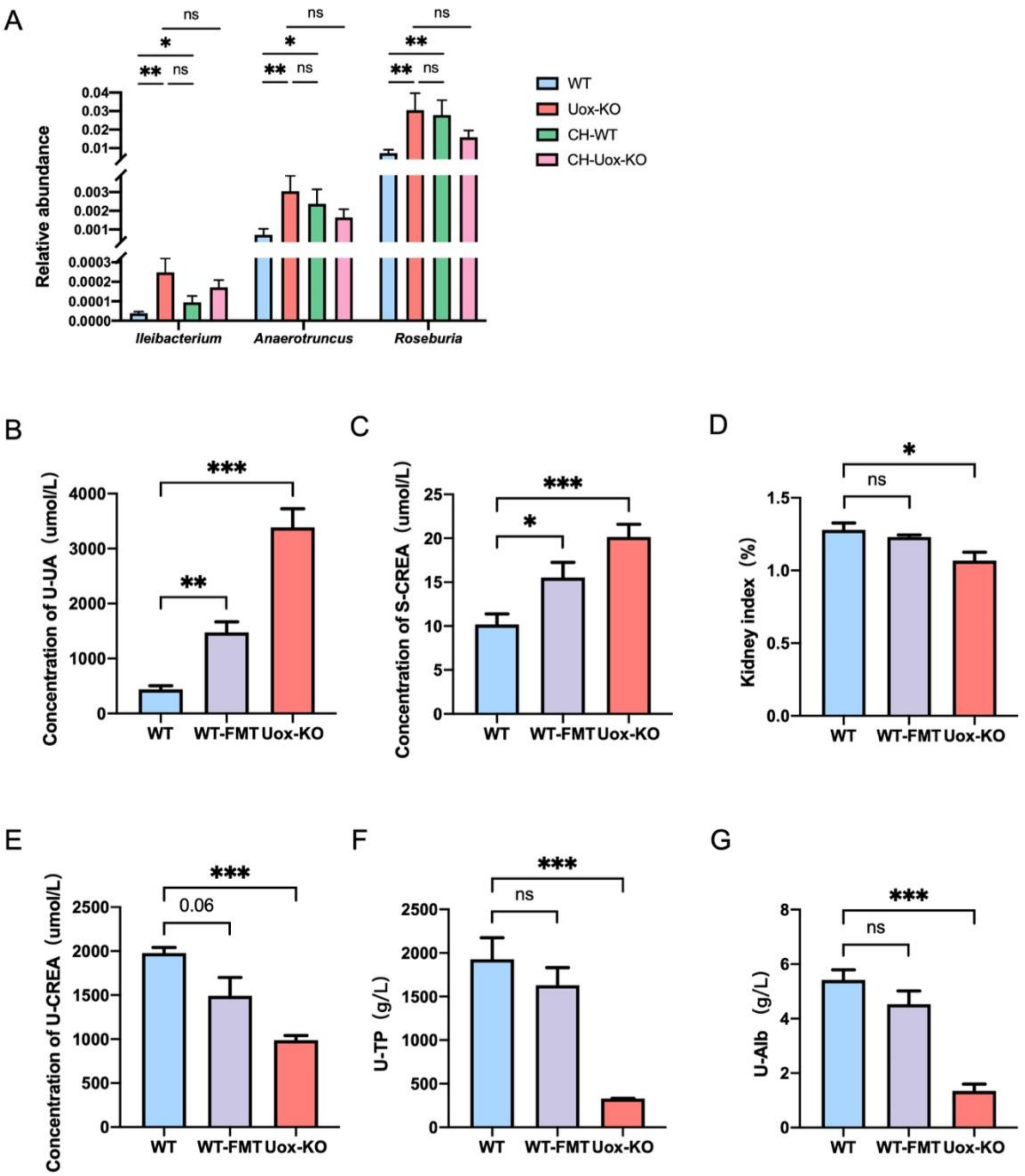

Figure S2

(A) The relative abundance of the strains that positively correlated to SUA in co-housed WT mice. “ns” represents not significant; * $p < 0.05$； ** $p < 0.01$.

(B-G) The level of UUA, SCr, Kidney index, UCr, U-TP and U-Alb in WT-FMT mice. “ns” represents not significant; * $p < 0.05$； ** $p < 0.01$; *** $p < 0.001$.

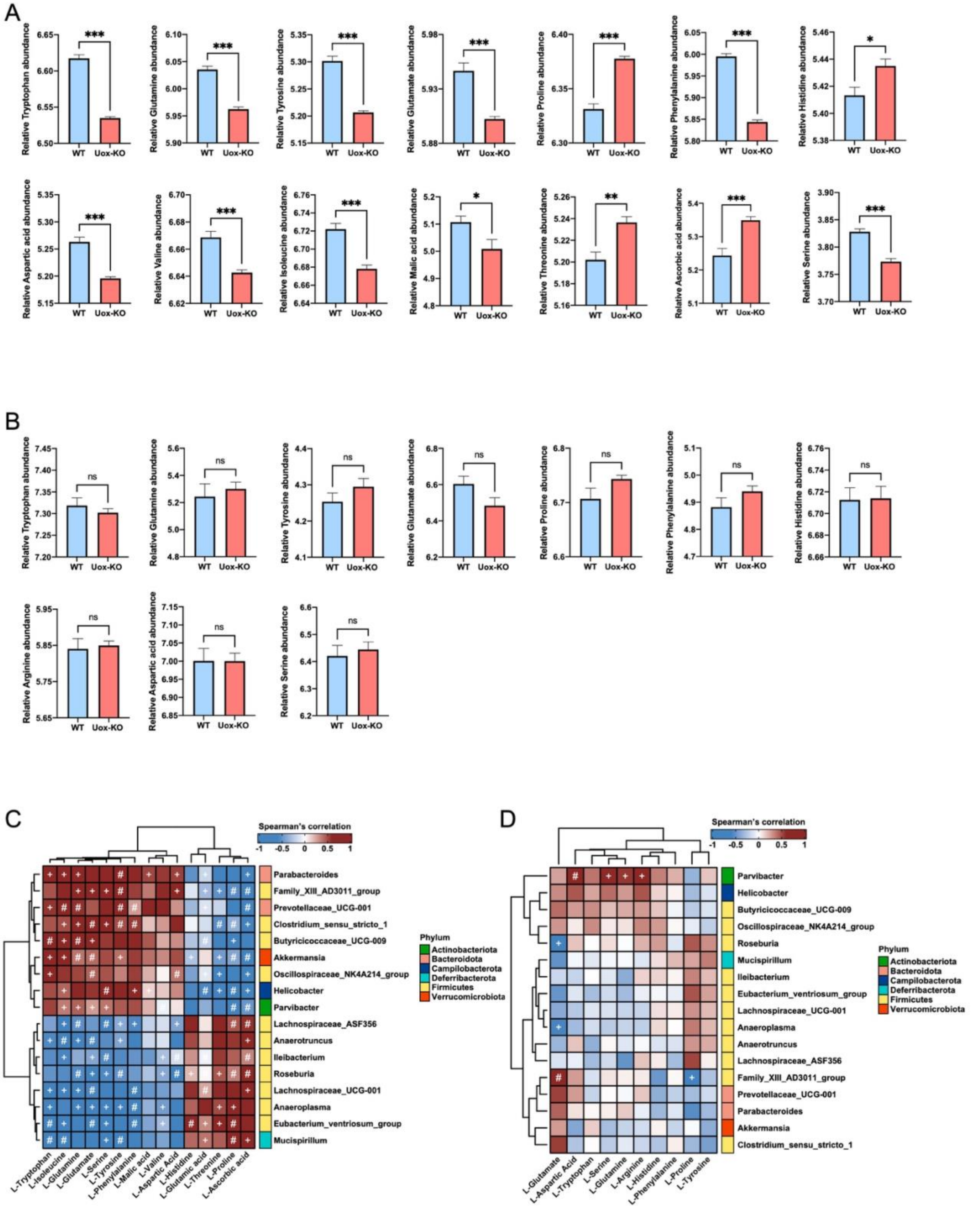
A
Relative Tryptophan abundance
Relative Glutamine abundance
Relative Tyrosine abundance
Relative Glutamate abundance
Relative Proline abundance
Relative Phenylalanine abundance
Relative Histidine abundance
Relative Aspartic acid abundance
Relative Valine abundance
Relative Isoleucine abundance
Relative Malic acid abundance
Relative Threonine abundance
Relative Ascorbic acid abundance
Relative Serine abundance
WT
Uox-KO
***
**
*
B
Relative Arginine abundance
ns
C
D
Spearman's correlation
-1
-0.5
0
0.5
1
Phylum
Actinobacteriota
Bacteroidota
Campilobacterota
Deferribacterota
Firmicutes
Verrucomicrobiota
Parabacteroides
Family_XIII_AD3011_group
Prevotellaceae_UCG-001
Clostridium_sensu_stricto_1
Butyricicoccaceae_UCG-009
Akkermansia
Oscillospiraceae_NK4A214_group
Helicobacter
Parvibacter
Lachnospiraceae_ASF356
Anaerotruncus
Ileibacterium
Roseburia
Lachnospiraceae_UCG-001
Anaeroplasma
Eubacterium_ventriosum_group
Mucispirillum
L-Tryptophan
L-Isoleucine
L-Glutamine
L-Glutamate
L-Serine
L-Tyrosine
L-Phenylalanine
L-Malic acid
L-Valine
L-Aspartic Acid
L-Histidine
L-Glutamic acid
L-Threonine
L-Proline
L-Ascorbic acid
L-Arginine

Figure S3

(A) The relative abundance of serum amino acids (N = 7 mice/group). * $p < 0.05$; ** $p < 0.01$; *** $p < 0.001$.

(B) The relative abundance of kidney amino acids (N = 7 mice/group). “ns” represents not significant.

(C) Spearman’s rank correlation analysis between discrepant microbial taxa and serum amino acids. Positive correlations are displayed in red and negative correlations in blue. The intensity of the color is proportional to the correlation coefficient. + $p < 0.05$; # $p < 0.01$.

(D) Spearman’s rank correlation analysis between discrepant microbial taxa and kidney amino acids. + $p < 0.05$; # $p < 0.01$.

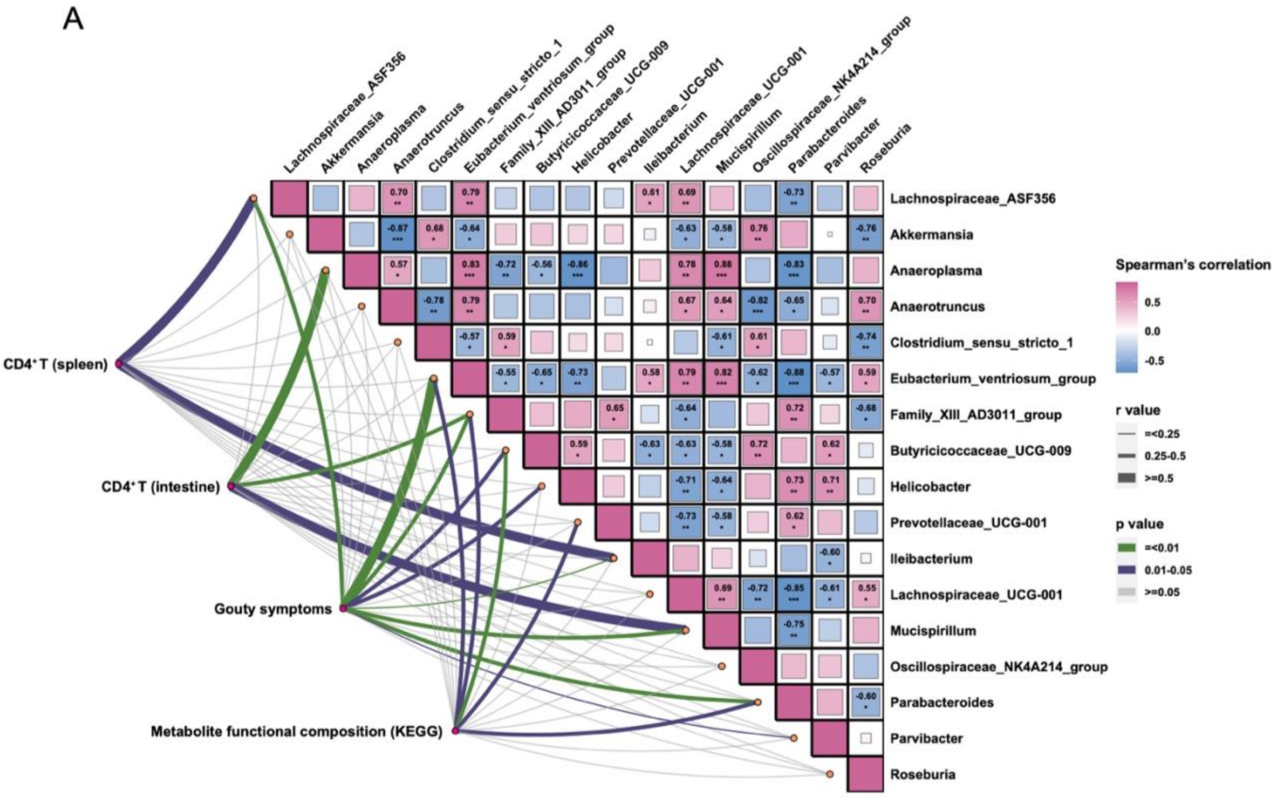

Figure S4

(A) Pairwise comparison of discrepant microbial taxa is shown with a color gradient signifying Spearman’s correlation coefficient. Diamonds size corresponds to the value of the correlation coefficients. Diamonds without a number denote the statistically non-significant pairwise correlations. Gouty symptoms, $CD4^+$ T cells and metabolite functional composition were related to discrepant microbial taxa by Mantel tests. Line width corresponds to the Mantel’s r statistic for the

corresponding distance correlations, and line color denotes the statistical significance based on 999 permutations. * $p < 0.05$; ** $p < 0.01$; *** $p < 0.001$.

References

1. Van Meurs JB, Pare G, Schwartz SM, Hazra A, Tanaka T, Vermeulen SH, et al. Common genetic loci influencing plasma homocysteine concentrations and their effect on risk of coronary artery disease. Am J Clin Nutr. 2013;98(3):668-76. doi: 10.3945/ajcn.112.044545

2. Cotlarciuc I, Malik R, Holliday EG, Ahmadi KR, Paré G, Psaty BM, et al. Effect of genetic variants associated with plasma homocysteine levels on stroke risk. Stroke. 2014;45(7):1920-4. doi: 10.1161/STROKEAHA.114.005208

3. Lofthouse EM, Brooks S, Cleal JK, Hanson MA, Poore KR, O'Kelly IM, et al. Glutamate cycling may drive organic anion transport on the basal membrane of human placental syncytiotrophoblast. J Physiol. 2015;593(20):4549-59. doi: 10.1113/JP270743

4. Console L, Scalise M, Mazza T, Pochini L, Galluccio M, Giangregorio N, et al. Carnitine Traffic in Cells. Link With Cancer. Front Cell Dev Biol. 2020;8:583850. doi: 10.3389/fcell.2020.583850

5. Nakayama A, Matsuo H, Shimizu T, Ogata H, Takada Y, Nakashima H, et al. Common missense variant of monocarboxylate transporter 9 (MCT9/SLC16A9) gene is associated with renal overload gout, but not with all gout susceptibility. Hum Cell. 2013;26(4):133-6. doi: 10.1007/s13577-013-0073-8

6. Gee HY, Jun I, Braun DA, Lawson JA, Halbritter J, Shril S, et al. Mutations in SLC26A1 Cause Nephrolithiasis. Am J Hum Genet. 2016;98(6):1228-34. doi: 10.1016/j.ajhg.2016.03.026

7. Long W, Panigrahi R, Panwar P, Wong K, D ON, Chen XZ, et al. Identification of Key Residues for Urate Specific Transport in Human Glucose Transporter 9 (hSLC2A9). Sci Rep. 2017;7:41167. doi: 10.1038/srep41167

8. Kimura T, Takahashi M, Yan K, Sakurai H. Expression of SLC2A9 isoforms in the kidney and their localization in polarized epithelial cells. PLoS One. 2014;9(1):e84996. doi: 10.1371/journal.pone.0084996

9. Wu KC, Reisman SA, Klaassen CD. Tissue distribution, hormonal regulation, ontogeny, diurnal expression, and induction of mouse cystine transporters Slc3a1 and Slc7a9. Free Radic Res. 2020;54(7):525-34. doi: 10.1080/10715762.2020.1812597

10. Wu D, Grund TN, Welsch S, Mills DJ, Michel M, Safarian S, et al. Structural basis for amino acid exchange by a human heteromeric amino acid transporter. Proc Natl Acad Sci U S A. 2020;117(35):21281-7. doi: 10.1073/pnas.2008111117

11. Bae M, Roh JD, Kim Y, Kim SS, Han HM, Yang E, et al. SLC6A20 transporter: a novel regulator of brain glycine homeostasis and NMDAR function. EMBO Mol Med. 2021;13(2):e12632. doi: 10.15252/emmm.202012632

12. Barnes SK, Eiby YA, Lee S, Lingwood BE, Dawson PA. Structure, organization and tissue expression of the pig SLC13A1 and SLC13A4 sulfate transporter genes. Biochem Biophys Rep. 2017;10:215-23. doi: 10.1016/j.bbrep.2017.04.005

13. Liu Y, Jin X, Ma Y, Jian Z, Wei Z, Xiang L, et al. Short-Chain Fatty Acids Reduced Renal Calcium Oxalate Stones by Regulating the Expression of Intestinal Oxalate Transporter SLC26A6. mSystems. 2021;6(6):e0104521. doi: 10.1128/mSystems.01045-21

14. Guo W, Li K, Sun B, Xu D, Tong L, Yin H, et al. Dysregulated Glutamate Transporter SLC1A1 Propels Cystine Uptake via Xc(-) for Glutathione Synthesis in Lung Cancer. Cancer Res. 2021;81(3):552-66. doi: 10.1158/0008-5472.CAN-20-0617

15. Wang W, Zou W. Amino Acids and Their Transporters in T Cell Immunity and Cancer Therapy. Mol Cell. 2020;80(3):384-95. doi: 10.1016/j.molcel.2020.09.006

16. Lee CC, Yang PK, Chen LC, Cheong ML, Tsai YL, Tsai MS. Associations between gene expression of magnesium transporters and glucose metabolism in pregnancy. J Formos Med Assoc. 2021;2:S0929-6646(21)00503-9. doi: 10.1016/j.jfma.2021.11.006
